# The Effect of an Altitude Training Camp on Swimming Start Time and Loaded Squat Jump Performance

**DOI:** 10.1371/journal.pone.0160401

**Published:** 2016-07-28

**Authors:** Amador García-Ramos, Igor Štirn, Paulino Padial, Javier Argüelles-Cienfuegos, Blanca De la Fuente, Carmen Calderón, Juan Bonitch-Góngora, Katja Tomazin, Boro Strumbelj, Vojko Strojnik, Belén Feriche

**Affiliations:** 1 Department of Physical Education and Sport, Faculty of Sport Sciences, University of Granada, Granada, Spain; 2 Department of Kinesiology, Faculty of Sport, University of Ljubljana, Ljubljana, Slovenia; 3 High performance Center of Sierra Nevada, Spanish Sport Council, Granada, Spain; Universidad Pablo de Olavide, Centro Andaluz de Biología del Desarrollo-CSIC, SPAIN

## Abstract

This study evaluated the influence of an altitude training (AT) camp on swimming start time and loaded squat jump performance. To accomplish this goal, 13 international swimmers (8 women, 5 men) were allocated to both the control (Sea Level Training, SLT) and experimental conditions (AT, 2320 m above sea level) that were separated by a one year period. All tests (15 m freestyle swimming start and loaded squat jumps with additional loads of 25%, 50%, 75%, and 100% of swimmers’ body weight) were performed before and after a concurrent 3-week strength and endurance training program prescribed by the national coach. Following the SLT camp, significant impairments in swimming start times to 10 (+3.1%) and 15 m (+4.0%) were observed (*P* < 0.05), whereas no significant changes for the same distances were detected following the AT camp (-0.89%; *P* > 0.05). Trivial changes in peak velocity were obtained during the loaded squat jump after both training periods (effect sizes: < 0.20). Based on these results we can conclude that a traditional training high—living high strategy concurrent training of 3 weeks does not adversely affect swimming start time and loaded squat jump performance in high level swimmers, but further studies are necessary to assess the effectiveness of power-oriented resistance training in the development of explosive actions.

## Introduction

Altitude training plays an important role in the physical preparation of athletes around the world [1]. Proof of this is that, worldwide, there are at least 22 altitude training centres located between 1000 and 3000 m above sea level (asl). Swimmers are amongst those athletes who use altitude training most often [2,3]. The High Performance Centre of Sierra Nevada is a popular centre for swimmers because of its location (2320 m asl; an optimal altitude according to Bonetti and Hopkins [1] and Wilber et al. [4]) and because it is one of the few altitude training centres in the world (the only one in Europe) with a 50-m pool. More than 300 swimmers of 12 different nationalities participated during 2015 in training camps at Sierra Nevada (usually 2–4 weeks duration) with the expectation of improving sea level performance.

In altitude training research using swimmers or other kinds of athletes as participants, most of the attention has been focused on endurance performance and related parameters (e.g., maximum oxygen consumption, total haemoglobin mass, etc.) [2,5]. The effectiveness of altitude training strategies in the development of endurance performance is generally accepted [1]. On the contrary, prolonged exposure to high altitude (> 5000 m asl) has been associated with a deterioration in lean mass [6,7] and its functional capacity [8–11]. However, there are no controlled studies examining the effect of typical altitude training routines, which are predominantly composed by endurance workouts, on the performance of explosive actions when the training is conducted at moderate altitude.

After an acute ascent to moderate altitude, previous studies have documented an increase in the velocity at which a determined absolute load can be lifted during resistance training exercises such as the bench press [12] and the loaded squat jump (LSJ) [13]. An increased activity of type II muscles fibers [14–16] or an increased excitability of the nervous system [17] could partially explain these results. However, the reduction in air resistance in hypobaric conditions has also been pointed out as a non-physiological mechanism [12,18]. Indeed, the air density affects performance of high velocity movement [19], and even though the speeds reached during the resistance training exercises is low (< 3 m∙s^-1^), reduced air density might also improve the performance of these movements [12,20], probably caused by its interaction with other factors such as the described above. Whilst doubts remain as to the principal mechanisms involved, it is possible that performing resistance training at altitude may offer benefits in the development of muscle power and the performance of explosive actions. In this regard, García-Ramos et al. [3] revealed significant increments in vertical jump height (≈ 7.2%) as well as in undulatory swimming start performance (≈ 2.8%) following a 2-week altitude training camp, but unfortunately this study did not include a control group (training at sea level).

Swimming start performance, commonly assessed as the time to 15 m [21–23], may be the specific swimming task most influenced by explosive force and muscular power [22]. The time to 15 m has been typically identified as a good predictor of overall race time in the four swimming strokes [24]. In addition, several studies have also evaluated start times to shorter distances (e.g., 5 and 10 m) to differentiate the variables that affect swimming start performance (block, flight, entry, glide and underwater propulsion phases) [23,25]. Developing a high horizontal impulse during the block phase in order to achieve the maximum possible horizontal take-off velocity is also a key factor in improving swimming start time [23,25]. Given the strong relationship between start time and overall race performance, and the clear influence of horizontal take-off velocity on swimming start performance, it seems appropriate to examine the effect of altitude training on these variables. Therefore, the main objective of the present study was to evaluate the influence of an altitude training camp on swimming start times and LSJ performance in high level swimmers.

## Materials and Methods

### Study design

A controlled trial was designed to assess the effects of a 3-week training camp held at moderate altitude on swimming start time and LSJ performance. To accomplish this goal, the same swimmers were tested under both control (Sea Level Training, SLT) and experimental conditions (Altitude Training, AT). The SLT camp was conducted at 295 m asl (Ljubljana, Slovenia) and the AT camp at 2320 m asl (High Performance Centre of Sierra Nevada, Granada, Spain). The SLT camp (February-March 2014) was conducted 1 year before the AT camp (February-March 2015), and all tests were performed before and after a 3-week training period. From the beginning of the study, the national coach was committed to maintaining the same training objectives for both SLT and AT conditions.

### Participants

The study population was comprised of 13 swimmers (8 women, 5 men) from the Slovenian national team. All swimmers were older than 16 years at the beginning of the study. The general characteristics of the swimmers at the outset of each training period are presented in Table 1. Swimmers were requested to include the LSJ exercise in their training schedule at least 1 year before the SLT camp. All swimmers were informed of the procedures to be utilized and signed a written informed consent form prior to investigation. For swimmers under 18 years old, written consent was obtained from their legal guardians. The study protocol adhered to the tenets of the Declaration of Helsinki and was approved by the University of Granada institutional review board.

**Table 1:** Descriptive characteristics of the study sample.

Variable	Sea Level Training			Altitude Training		
	Men (n = 5)	Women (n = 8)	All (n = 13)	Men (n = 5)	Women (n = 8)	All (n = 13)
Age (years)	18.7±3.7	17.7 ± 3.4	18.1 ± 3.4	19.7±3.7	18.7±3.4	19.1±3.4
Height (cm)	180.7±2.6	167.1 ± 5.4	172.3 ± 8.2	181.4±2.6	168.4±5.6	173.4±8.0
Body mass (kg)	70.7±6.3	57.5±5.0	62.6±8.5	72.3±4.2	58.4±4.8	63.7±8.3
FPS	807.4±63.8	793.5±86.8	798.8±76.2	781.4±65.3	808.3±81.8	797.9±74.2

FPS, Fina Point Score (data from 2012 and 2013 for sea level and altitude training, respectively).

### Testing procedures

The participants were familiarized with the test procedures before the measurements were taken and tests were conducted within a single day and at the same time of day for each individual swimmer. The following measurements were taken in a sequential order:

#### 1. Swimming start

**Fig 1:**
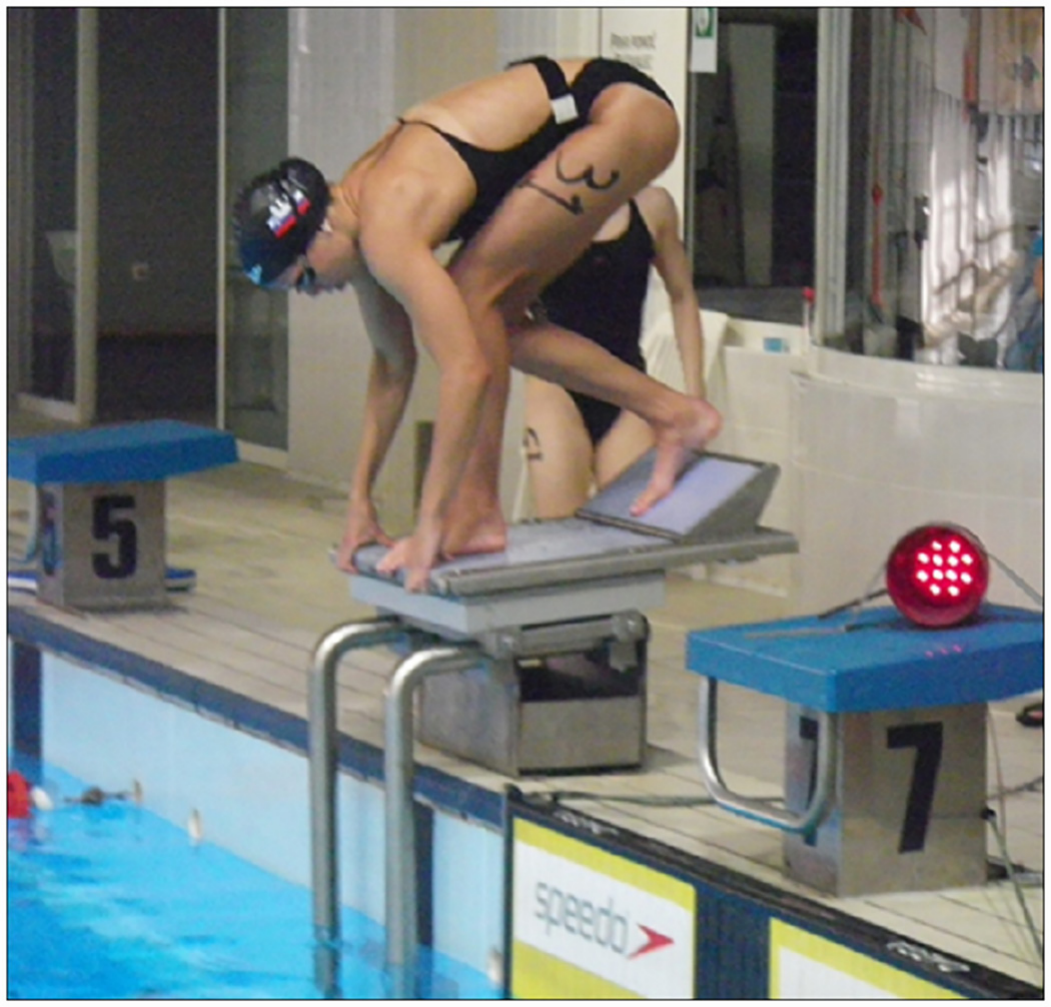
Swimmer ready to perform the freestyle track start. Note: The individual in this picture has given written informed consent (as outlined in PLOS consent form) to publish this photograph.

After completing a standard warm–up based on their pre–race routine, swimmers were instructed to perform two freestyle track starts to a distance further than 15 metres to ensure representative values of the time to 15 metres [21,23]. A standardised starting procedure was used. Swimmers waited on the starting block and when they were ready, a tester gave the command ‘‘take your mark”, before a sound was made by shutting a clapperboard to signal the start of the trial (Fig 1).

**Fig 2:**
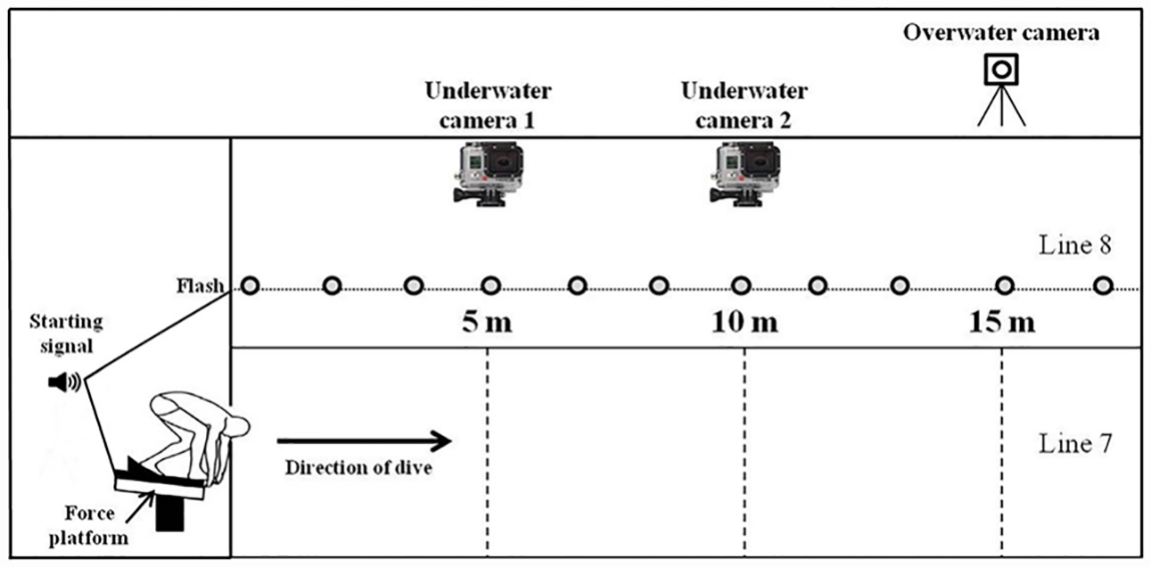
Graphical representation of the measurement equipment used to analyze swimming start performance. Note that another starting flash (not depicted) was positioned at the other poolside perpendicular to the overwater camera.

Two underwater cameras (GoPro Hero 3, 100 fps) (GoPro,Inc. San Mateo, California, USA) and an overwater camera (Casio Exilim Pro EX-FX1, 300 fps) (CASIO Computer CO., Ltd. Tokyo, Japan) were set up such that their optical axes were perpendicular to the direction of swimming at 5, 10, and 15 m from the starting position, respectively [23]. The shutting of the clapperboard, in addition to emitting the acoustic starting signal, synchronously activated a light system that was extended from the beginning to the end of the swimming pool at 1 metre depth. Each camera was positioned to record at least one of the LEDs. When processing the data, the first frame in which the LEDs were switched on was used to determine the zero time of the video recordings. A 2D reference system was built with non-elastic lead ropes hooked onto the roof of the swimming pool at the distances analyzed (5, 10, and 15 m). An overview of the measurement equipment used in this study is depicted in Fig 2.

The times to 5, 10 and 15 metres were defined as the time elapsed from the starting signal until the swimmer’s head crossed the 5, 10, and 15 metre marks, respectively. The analysis was done by the Ultimate Pen Software (St Paul, Minnesota, USA) which allowed us to play the video image as well as to plot the spatial references determined from the 2D reference system. The implementation of a routine (Script) in the Filemaker Pro v.12 software (Santa Clara, California, USA) enabled the time code of the video image to run with QuickTime Player v7 (Cupertino, California, USA) and set this time in its specific database field for further processing. The start with the lowest time to 15 m was selected for subsequent analysis.

To measure ground reaction force during the start, a portable force plate (Kistler 9253A11, Winterthur, Switzerland) was put on a custom-made stand with an angle of 7° to the horizontal and a custom-made steel starting block (identical to OSB11) was mounted on top of the force plate. Horizontal and vertical forces were collected with a sampling rate of 1000 Hz using custom–programmed data collection software, and were used to determine the horizontal take-off velocity following standard procedures of calculation [22,23]. The horizontal take-off velocity was selected because it has been identified as the most determinant variable of the push-off phase in terms of overall start time [23,25].

#### 2. Loaded squat jump (LSJ)

Prior to testing, swimmers completed a 10-min standardized warm–up based on jogging, joint mobility, dynamic stretching, 6 jumps without additional weight, and 1 set of 5 LSJ with an unloaded Smith machine bar (16 kg). Thereafter, an incremental loading test using the SJ exercise with additional loads of 25%, 50%, 75%, and 100% of swimmers’ body weight was conducted with a Smith machine. Two attempts per load were performed. One min of rest was allowed between the same load trials, and 5 min between trials with different loads. The general characteristic of the LSJ technique has been described elsewhere [26].

A linear velocity transducer (T-Force System; Ergotech, Murcia, Spain), validated by Sánchez-Medina and González-Badillo [27], was attached to the bar to record its vertical instantaneous velocity at a sampling frequency of 1000 Hz. The peak velocity was the dependent variable analyzed. Only the jump with the highest peak velocity of each load was considered for further analysis.

### Training Procedures

The study was carried out during the second macrocycle (short-course season) of the year (February-march 2014 and 2015). The intervention period comprised a mesocycle of 3 weeks during the general preparation phase. Accordingly, it was a condition of participation that the relative training load would not substantially change during the 3-week study phase between years to allow the full assessment of the training intervention without such a confounding factor. To minimize the influence of fatigue, coaches were asked to reduce the training load the day before to the assessment days.

Individualized training plans were developed by the swimmers’ coaches, each very experienced in AT. They implemented the training program according to their own experience, swimmer’s fitness level, and individual response to altitude. Typically, training schedules included two pool sessions and a dry-land workout six days per week. Throughout the entire duration of the training period, the main coach of the national team (for pool training sessions) and the fitness coach (for dry-land training sessions) were responsible for filling in the training diary of each swimmer. Pool training was described in terms of time and distance swum. Dry-land sessions were described by reporting the main purpose and the content of training. The main purpose was expressed by the selection of a code: 1 for sessions oriented to developing maximum strength; 2 for explosive strength; 3 for endurance strength; 4 for conditioning; 5 for cardiovascular activities; and 6 for range of motion and flexibility. For codes 4 to 6, a brief description of the content was also incorporated. For codes 1 to 3, additional information, such as the number and description of exercises, sets, repetitions per set, load, rest between sets, and speed of the movement, was also detailed. Maximum strength training sessions involved 6–8 exercises in which 3–4 sets of 6–12 repetitions at 70–85% of the 1-repetition maximum (1RM) were performed, with 2–5 min of rest. During endurance strength training sessions, sets of 20 repetitions or maximum repetitions in 20-40s sets were performed using a load of 30–50% of 1RM, followed by < 1 min of rest. Different variants of the squat (front squat, deep back squat, Bulgarian split squat, etc.), deadlift, leg flexion and extension, and hip thrust were the most common lower limb exercises employed by the swimmers.

Additionally, within 30 min after each training pool or dry-land session, a category scale (0–10) of ratings of perceived exertion (C-RPE10) [28] was undertaken to assess training intensity.

### Statistical analyses

Data are presented as means and standard deviations (SD). Reliability of the measurements was calculated by determining the intraclass correlation coefficient (ICC) and the 90% confidence interval (90% CI) using a custom spreadsheet [29]. A two-way (training condition [SLT and AT] x test [pretest and postest]) repeated measures ANOVA was used to determine the differences at baseline between both training periods and the training-related effects for each dependent variable analyzed. When significant F values were obtained, pairwise differences between means were identified using Bonferroni post hoc procedures. Effect sizes (ES) using Cohen’s *d* ([posttest mean—pretest mean] / pretest SD) and percentage differences ([posttest mean—pretest mean] / pretest mean × 100) were also calculated. The percentage changes after each training period were used to compare training-related effects between SLT and AT through paired samples t-tests. Significance was set at *P* < 0.05. The criteria to interpret the magnitude of the ES were as follows: <0.2 = trivial, 0.2–0.6 = small, 0.6–1.2 = moderate, 1.2–2.0 = large, and >2 = very large [30]. All statistical tests were performed using the software package SPSS (version 20.0: SPSS, Inc., Chicago, IL, USA).

## Results

Throughout the camp, daily average pool-sessions were no different between the SLT and AT period, with a total distance swum of 8853 ± 2430 m and 10147 ± 3651 m (*P* = 0.538), total time of 106.9 ± 11.5 min and 113.2 ± 2.0 min (*P* = 0.078), and C-RPE10 scale of 5.74 ± 0.97 and 5.72 ± 0.29 (*P* = 0.824), for SLT and AT respectively.

Dry-land sessions were generally oriented to strength and conditioning. Relative intensity of dry-land session was of 6.16 ± 1.11 vs 5.45 ± 0.62 for SLT and AT, respectively (*P* > 0.05). There was no difference between the total number of resistance training sessions performed in both training periods (code 1: 5.91 ± 2.43 and 5.31 ± 0.65 [*P* = 0.479], and code 3: 4.67 ± 2.08 and 4.31 ± 0.63 [*P* = 0.634] for SLT and AT, respectively. Additionally, an average of two dry-land sessions of code 4 and two of code 5 were completed during the SLT period, while during AT, one session of code 2 and five of code 4 were performed.

**Fig 3:**
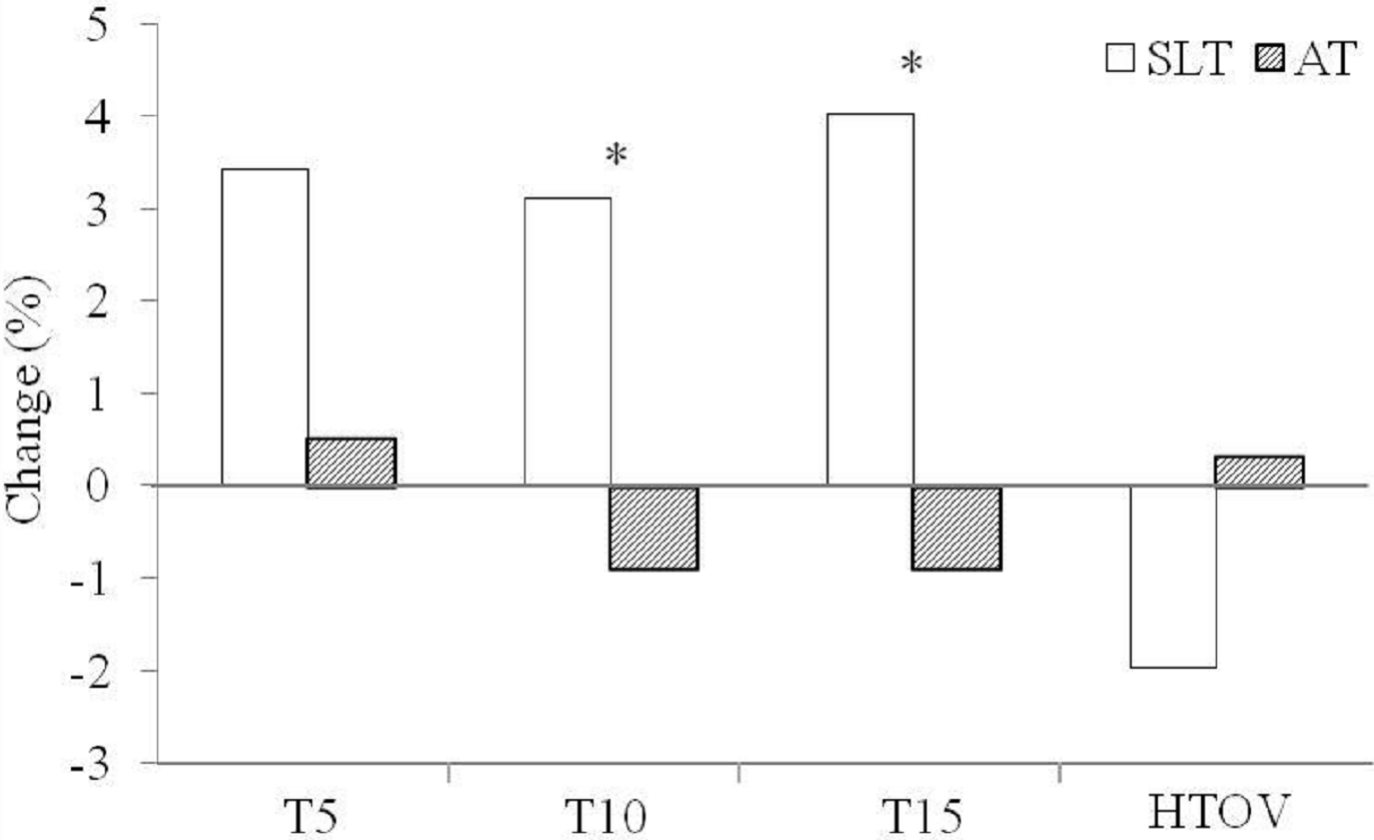
Percent changes in swimming start performance after the sea level training (SLT) and altitude training (AT) periods. T5, Time to 5 m; T10, Time to 10 m; T15, Time to 15 m; HTOV, Horizontal take-off velocity. *, Significant differences between percent changes (*P* < 0.05). Standard deviations have been omitted for clarity but are contained in Table 2.

The reproducibility of the swimming start skill was confirmed (ICC: 0.90–0.97). At baseline, the time to 15 m was significantly better in the SLT than in the AT conditions (*P* = 0.009; ES = 0.38), whereas no significant differences were obtained for the times to 5 and 10 m. After 3 weeks of SLT, swimming start times became significantly slower; however, there were no changes in start times after 3 weeks of AT (Table 2; Fig 3). The horizontal take-off velocity did not change in any of the training periods.

**Table 2:** Pre to post changes in swimming start performance after 3-weeks of sea level (SLT) and altitude training (AT).

	Sea level training camp				Altitude training camp			
	Pre	Post	% of change	ES	Pre	Post	% of change	ES
T5 (s)	1.63 ± 0.18	1.66 ± 0.15	3.43 ± 4.97	0.21	1.68 ± 0.14	1.69 ± 0.14	0.51 ± 3.14	0.06
T10 (s)	4.37 ± 0.42	4.47 ± 0.39*	3.11 ± 2.48	0.24	4.45 ± 0.42	4.41 ± 0.43	-0.89 ± 2.53¥	-0.09
T15 (s)	7.26 ± 0.51	7.54 ± 0.61*	4.02 ± 3.26	0.54	7.46 ± 0.54	7.40 ± 0.59	-0.89 ± 2.78¥	-0.12
Horizontal take-off velocity (m·s^-1^)	4.28 ± 0.25	4.21 ± 0.30	-1.96 ± 3.62	-0.29	4.24 ± 0.31	4.28 ± 0.25	0.33 ± 2.35	0.14

T5, Time to 5 m; T10, Time to 10 m; T15, Time to 15 m; ICC, Intraclass correlation coefficient; 90% CI, 90% confidence interval; % of change, Percent difference ([Post mean—Pre mean] / Pre mean × 100); ES, effect size ([Post mean—Pre mean] / SDpre);

*, Significant differences between pretest and postest (*P* < 0.05).

^¥^, Significant differences between percent changes.

**Fig 4:**
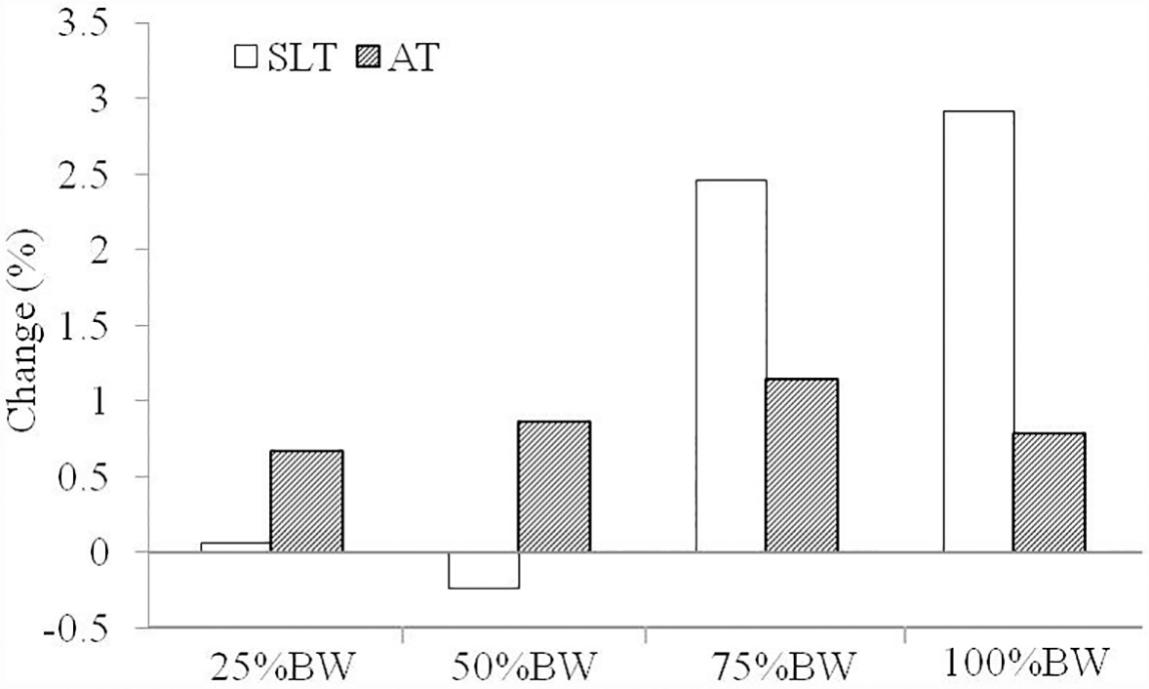
Percent changes in loaded squat jump performance (peak velocity) after the sea level training (SLT) and altitude training (AT) periods. BW, Body weight. Standard deviations have been omitted for clarity but are contained in Table 3.

High reliability was observed for the peak velocity achieved with the 4 loads evaluated (ICC: 0.86–0.94). At baseline, LSJ peak velocity was higher for AT compared to the SLT with the 4 loads analyzed (*P* < 0.01; ES = 0.59–0.67). Trivial changes in peak velocity were obtained during the LSJ after each training period (effect sizes: < 0.20), with no significant differences between experimental conditions (Table 3; Fig 4).

**Table 3:** Pre to post changes in loaded squat jump peak velocity after 3-weeks of sea level (SLT) and altitude training (AT).

Load	Sea level training camp				Altitude training camp			
	Pre (m·s^-1^)	Post (m·s^-1^)	% of change	ES	Pre (m·s^-1^)	Post (m·s^-1^)	% of change	ES
25% BW	2.15 ± 0.30	2.15 ± 0.28	0.06 ± 5.48	-0.01	2.33 ± 0.28	2.34 ± 0.26	0.67 ± 4.27	0.04
50% BW	1.90 ± 0.24	1.90 ± 0.23	-0.24 ± 4.69	-0.03	2.06 ± 0.27	2.06 ± 0.17	0.86 ± 7.28	0.01
75% BW	1.63 ± 0.25	1.66 ± 0.21	2.46 ± 6.01	0.12	1.79 ± 0.22	1.80 ± 0.19	1.14 ± 5.74	0.07
100% BW	1.44 ± 0.22	1.48 ± 0.19*	2.91 ± 3.38	0.17	1.57 ± 0.20	1.57 ± 0.20	0.78 ± 6.66	0.04

BW, Body weight; ICC, Intraclass correlation coefficient; 90% CI, 90% confidence interval; % of change, Percent difference ([Post mean—Pre mean] / Pre mean × 100); ES, effect size ([Post mean—Pre mean] / SDpre);

*, Significant differences between pre and post (*P* < 0.05).

## Discussion

This study was designed to investigate the effectiveness of an altitude training camp on swimming start and LSJ performance. Both training periods caused similar small changes in the analyzed variables. However, it should be noted that the training regime followed by the swimmers, which was strongly oriented towards improving endurance capacity, did not allow us to identify whether or not power-oriented AT might genuinely enhance the contractile force of the muscles and consequently the performance of explosive actions. Nevertheless, the results of the present study suggest that a training high—living high strategy of 3 weeks at 2320 m asl does not have adverse effects on muscular function, even if swimmers do not focus their training solely on improving force and power.

Traditionally, prolonged exposure to altitude has been associated with a deterioration in lean mass [6,7] and its functional capacity [8–11]. Different factors such as an insufficient energy intake [31,32], a reduced training stimulus [12], or even the negative effect of hypoxia itself on protein metabolism [33] have been proposed as being responsible of these impairments. However, it should be considered that the vast majority of adverse effects of chronic hypoxia effecting muscle size and strength/power adaptations have been documented at an altitude above 5000 m asl; far higher than the 2000–2500 m generally recommended and where the present study was conducted [1,4].

This is one of the first studies evaluating the performance of explosive actions (swimming start time and LSJ) after an altitude training camp held at terrestrial moderate altitude (2320 m asl). Both training periods promoted similar changes in the analyzed variables. These results have important applications in the field of altitude training as they indicate that 3-weeks of a training high—living high strategy does not constitute a negative stimulus on muscular function. Therefore, it would seem unnecessary for swimmers to be concerned about the loss of lean mass and its functional capacity when living and training at moderate altitude.

The changes in swimming start time to 5 and 10 m observed after the AT camp represented a little but significant improvement over the SLT period. However, rather than attributing this to the effect of the AT training camp, we should acknowledge that this result was mainly caused by the significant impairment in swimming start time observed after the SLT period. The time to 5 m, in concordance with the results obtained for the horizontal take-off velocity and LSJ performance, did not differ between measurements. These results suggest that the differences between the training periods were caused by the swimming part of the start, which mainly depends on the efficiency of underwater legs kicking and maintaining the optimal hydrodynamic body position.

The training regime followed by the swimmers was excessively oriented towards the improvement of endurance capacity which could explain the results of the present study since it is known that concurrent endurance training attenuates strength training responses [34]. In this regard, Häkkinen et al. [35] reported that concurrent strength and endurance training leads to interference in explosive strength development. Therefore, since explosive strength is paramount for the actions analyzed in the present study (LSJ and swimming start) [22], it is logical that the improvements recorded in LSJ performance were not very pronounced. Similar weak enhancements in LSJ were produced after each training period (SLT and AT). These results confirm that a typical 3-week concurrent strength and endurance training program performed at terrestrial altitude does not have adverse effects on vertical jump performance in high level swimmers.

While the majority of studies carried out with swimmers at altitude have been focused on parameters related to aerobic metabolism [2], this is the first study examining the effect of a traditional AT camp on the performance of explosive actions. The main conclusion of the present study is to report that the performance of explosive actions is not impaired after a stay of 3 weeks at terrestrial altitude even if swimmers do not change their strength training routine in an attempt to improve these functions. However, it would be necessary for future studies to carry out resistance training programs exclusively designed to develop maximum and explosive strength to further explore the applications of AT in the field of strength and conditioning. Due to logistical constraints it was not feasible to split the national team in 2 groups to counterbalance the order of the training interventions. To minimize the impact of this potential limitation only the swimmers older than 16 years at the beginning of the first phase of the study were included in the analysis. The younger swimmers were excluded because the accentuated year-to-year variability in the biological maturation generally observed at these ages could have a major effect in our results.

## Conclusions

A traditional 3-week concurrent strength and endurance training program performed at terrestrial altitude does not adversely affect swimming start time and LSJ performance in high level swimmers. These results confirm that swimmers should not be excessively concerned about the deterioration of muscular function when they take part in AT camps at moderate altitude. Power-oriented resistance training must be performed at terrestrial altitude to examine further whether altitude training has an additional benefit on the development of explosive actions when compared to training at sea level.
